# The Influence of Diatomite Addition on the Properties of Geopolymers Based on Fly Ash and Metakaolin

**DOI:** 10.3390/ma17102399

**Published:** 2024-05-16

**Authors:** Marek Nykiel, Kinga Korniejenko, Kinga Setlak, Mykola Melnychuk, Nina Polivoda, Barbara Kozub, Maria Hebdowska-Krupa, Michał Łach

**Affiliations:** 1 Faculty of Materials Engineering and Physics, Cracow University of Technology, Warszawska 24, 31-155 Cracow, Poland; marek.nykiel@pk.edu.pl (M.N.); kinga.setlak@pk.edu.pl (K.S.); barbara.kozub@pk.edu.pl (B.K.); maria.hebdowska-krupa@pk.edu.pl (M.H.-K.); michal.lach@pk.edu.pl (M.Ł.); 2 Department of Materials Science, Lutsk National Technical University, Lvivska 75, 43000 Lutsk, Ukraine; m.melnychuk@lntu.edu.ua (M.M.); palivoda.nino@gmail.com (N.P.)

**Keywords:** geopolymer, diatomite additive, fly ash, metakaolin

## Abstract

Geopolymer materials, considered to be an alternative to Portland cement-based concretes, can be produced from various types of waste aluminosilicate raw materials. This article presents the results of research related to the use of diatomite as an additive in geopolymers. The results of testing geopolymer composites with 1%, 3%, and 5% additions of diatomite with a grain size of 0–0.063 mm after and without thermal treatment were presented. This article presents the physical properties of the diatomite additive, the morphology of diatomite particles SEMs, thermal analysis, and compressive strength test results. In this research, diatomite was treated as a substitute for both fly ash and metakaolin (replaced in amounts of 1 and 3%) and as a substitute for sand introduced as a filler (in this case, 5% of diatomite was added). As a result of this research, it was found that the addition of diatomite instead of the main geopolymerization precursors in amounts of 1 and 3% had a negative impact on the strength properties of geopolymers, as the compressive strength was reduced by up to 28%. The introduction of crushed diatomite instead of sand in an amount of 5% contributed to an increase in strength of up to 24%.

## 1. Introduction

Geopolymers are modern, environmentally friendly, and inorganic aluminosilicate materials that are considered an alternative to Portland cement [1,2]. Because of their high mechanical properties, the main area of potential application is civil engineering, especially the most demanding applications in construction industries [1,3]. Except for the excellent strength properties, they are also characterized by fire-resistance and high thermal stability, widening their area of application; they can also be utilized as lightweight materials for thermo-isolation purposes [3,4,5]. In addition, to increase their excellent mechanical properties, various types of additives have been tested as reinforcements [6,7,8]. One of the possibilities seems to be diatomite. According to chemical composition, it contains about 80–90% SiO_2_, so it should be useful in the geopolymerization process [9,10].

The first study that confirmed the theoretical possibility of applying diatomite was conducted by Gupta et al. [11]. They found a non-hydrolysable geopolymer additive in fossil leaves from the Ardèche diatomite in France [11]. These are grounds for stating that diatomite can be used in the geopolymerization process. This was confirmed by Sinsiri et al. [12], who replaced fly ash with diatomite in geopolymer mortar. However, the addition of diatomite reduced the geopolymer’s compressive strength and modulus of elasticity, but it improved other material characteristics. This research confirms that joining diatomite with fly ash allows researchers to obtain geopolymer mortars with reduced weight, improved workability, and enhanced strain capacity [12]. The same team also provided research for high-calcium fly ash geopolymer, where diatomite was applied as an additive in amounts of 0, 10, 20, 30, and 40% [13]. The results show negative effects such as a reduction in mechanical properties (strength and modulus of elasticity), increase in the cost of the final material, and energy consumption throughout the whole process; they also demonstrated some positive influences, including a delay in the setting time of fresh geopolymer paste, a decrease in the material’s density, and an increase in strain capacity, including cracking reduction [13].

Arbi et al. [14] explained this phenomenon and compared the mixtures based on diatomite with other geopolymers based on blast furnace slag (BFS). The reaction was achieved using two different activators: a moderately alkaline medium (Na_2_SO_4_) and a highly alkaline medium (NaOH) [14]. In a moderately alkaline medium, they found that activating the blend using BSF creates C–(A)–S–H gel and sulphoaluminates (ettringite and phase U), whereas diatomite-based mixtures create only CAC hydration products (ettringite and phase U). This indicates a lack of proper reaction. In a highly alkaline medium, BSF-based material creates (N,C)–A–S–H gel, katoite, and carbo aluminates (hydrotalcite, C4AcH_11_). Under the same conditions, diatomite shows only partial reactivity, caused by the scant availability of the majority of elements (Si, Al, and Ca), which does not allow for obtaining a sufficient amount of cementitious gel [14]. This research presented a novel approach to the use of diatomite in the geopolymerization process. It was no longer investigated as a basic feedstock but rather as a valuable additive in the geopolymerization process. This approach was also applied by Santos et al. [15] who incorporated up to 1.5% diatomite into a geopolymer matrix and, as a result, observed an improvement in mechanical properties, changes in microstructure, and the retention of water in the paste (decrease in the filtrate volume) [15]. 

The same aim, reducing alkaline solution consumption in geopolymers through diatomite addition, was the main motivation of the research provided by Şahbudak [16]. Their results show that geopolymers with a very small amount of sodium hydroxide (1M) supplemented with diatomite (10 wt.%) can be applied as construction materials with reasonable strength properties—18 MPa compressive strength [16]. Furthermore, some more recent research is focused on this topic. Feltaous et al. [17] confirmed previous investigations and clearly pointed out that the new approach has great potential to reduce the cost and environmental impact of geopolymers [17]. Moreover, Coelho et al. [18] show that geopolymerization reactions using diatomite as a silica source allow the development of silicate-free geopolymers with enhanced performance at low temperatures [18]. An investigation into different silica sources was also conducted by Chen and Li [19], who developed their research in terms of obtaining red mud geopolymer for use in slope protection applications. They confirmed that a 5% addition of diatomite has a positive effect on plant growth [19].

A similar approach was presented by Bagci et al. [20], who applied diatomite as a fumed silica alternative in metakaolin-based geopolymers [20]. The obtained findings show such possibilities. However, the flexure strength of the materials with diatomite was slightly lower; the compressive strength and Weibull modulus were significantly higher [20]. The improvement in compressive strength with the increase in diatomite (up to 20%) also confirms the findings of Thammarong et al. [21] and Rocha et al. [22]. Font et al. [23] confirmed the use of diatomite as an alternative silica source. They also showed that the calcination process can improve the properties of diatomite [23]. 

Kljajević et al. [24] provided comparative research on the water contact angle of several geopolymers, including diatomite-based geopolymers. The best values, the smallest porosity, and the highest hydrophobicity were achieved for geopolymers synthesized from diatomite (compared with those synthesized from bentonite and kaolinite) [24]. Kljajević et al. [25] also investigated electrical conductivity for the same compositions. In this case, the best conductivity was revealed for metakaolin-based geopolymer [25].

Thammarong et al. [26] investigated the influence of the calcination temperature of diatomite on the final properties of the geopolymer. They applied temperatures in the range 500–1200 °C. The best results were obtained for 700 °C, the lowest density, and the highest mechanical properties [26]. However, it is worth noticing that the temperature required for other diatomite deposits to obtain desirable properties of the final material can be slightly different. 

As an alternative to the traditional geopolimerization process, Hassan et al. [27] investigated the possibility of designing a so-called one-part geopolymer. His conception is based on pre-mixing CaCO_3_-rich wood biomass ash from the brick industry with diatomite and different proportions of sodium hydroxide [27]. This composition required just adding water to create a geopolymer material that is a competitive alternative to Portland cement. The results show high potential for applying this mixture, including high compressive strength, developing with time. The enhancement mechanism is connected to the continuation of the dissolution of diatomite ingredients—aluminosilicate and the formation of chemical phases such as calcium silicate hydrate and calcium aluminosilicate hydrate, which improve the strength of the material [27].

The main area of interest investigated by Liguori et al. was geopolymer foams [28]. In this research, the organic–inorganic hybrid foams were modified by replacing part of the composition that included metakaolin with diatomite. The results show the influence of diatomite on the microstructure and mechanical behavior. The best results were achieved by replacing half of the metakaolin with diatomite [28]. The research on geopolymers formed using diatomite was continued by Yen et al. [29]. This team prepared a lightweight material–foamed geopolymer using metakaolin and diatomite powders as raw materials, characterized it, and investigated it at high temperatures (800, 1000, and 1200 °C) [29]. They found that the diatomite addition increases the density of the materials [29]. They also revealed that the materials’ microstructure changed according to temperatures—it decreased the open porosity and at the 1200 °C the structure changed into a leucite crystal phase [29]. Further research on the foamed materials has been conducted by Brudny et al. [30]. In this case, the non-calcinated diatomite was used as a replacement for fly ash in amounts of 5%, 10%, and 50%. The research shows that the addition of diatomite increases thermal properties while decreasing density and mechanical properties [30]. The obtained material has properties that allow for its potential application as a thermo-isolating material in the construction industry [30]. The foamed geopolymers based on blast furnace slag with attapulgite and diatomite addition were investigated by Kaplan et al. [31]. This research focused mainly on the influence of attapulgite on material properties [31]. They show the potential of diatomite-application-formed composition as an alternative to brick and non-structural wall elements, including in elevated temperatures [31].

The geopolymer’s temperature resistance was also investigated by Özsoy et al. [32]. This research focused on the influence of diatomite on material properties in elevated temperatures—300, 600, and 900 °C [32]. The diatomite was added in amounts of 1, 2, 3, 4 and 5%. The results show that, according to the mechanical properties, the best results were obtained for 1% and 2%; in these cases, diatomite addition increased the flexural and compressive strengths compared to the reference sample [32].

Salam et al. [33] and Abukhadra et al. [34] developed geopolymer compositions with diatomite for application outside the construction industry. They noticed that this kind of material has a catalytic property and could be applied in wastewater treatment, including radioactive elements such as strontium Sr (II) [33]. In further studies, they also developed this composition for the purification of groundwater and sewage water from such ions as phosphate and ammonium, which can be applied against the eutrophication process [35] as well as in a system of water desalination [36] based on the same technology—geopolymer-based compositions. 

Analysis of the current state of knowledge allows for the formulation of potential research areas. Firstly, some previous research has been conducted using diatomite additives; the authors used diatomite with a large particle size, not a diatomite fume, as an additive. Potentially, advancements made using the smallest fraction of diatomite can result in the greatest ecological and economic benefits, because it is this fraction that is considered to be waste when mining diatomite. Secondly, most scientists in the field of research have tested relatively large amounts of diatomite additive, but the latest investigation conducted by Özsoy et al. [32] shows that a small amount of diatomite can work better. It is important to take into consideration previous experiences with silica fume, which show that micro additives work better [7,8]. Previous research has also investigated a diatomite source not explored previously for geopolymer synthesis and analysis, under two different conditions—delivery conditions and after calcination at 850 °C [9,10]. The main goal of this article is to show the possibility of using diatomite fume in geopolymeric materials. In the presented research, geopolymers were made based on fly ash and sand or metakaolin and sand in a weight ratio of 50/50% with 1% and 3% additions of diatomite dust with a grain size of 0–0.063 mm and with a variable ash to sand ratio in which some of the sand was replaced by diatomite. Previous research has indicated that the addition of diatomite to foamed insulating geopolymers contributes to the improvement of strength parameters [30]. Similarly, when diatomite was used in geopolymers based on low-quality ashes from lignite combustion, an improvement in strength properties was achieved in some cases [9]. Although diatomite has many applications, research has been carried out on the possibility of using waste from its extraction because the source of diatomite is not of the highest quality. Diatomites extracted in Poland are significantly contaminated and have not yet found wide application beyond their use as sorbents of petroleum substances and in agriculture or animal breeding. The possibility of using diatomite waste or its utilization in geopolymer binders may have positive effects on the environment, and the presented research results may encourage other scientists to look for other more effective ways of using diatomite. The novelty of the work is the approach in which diatomite was treated as a substitute for both fly ash and metakaolin in amounts of 1 and 3%, and tests were also carried out using a larger share of 5% of replaced sand. The presented research results are more clear and easier to apply because they directly show that waste diatomite can be successfully used as a substitute for fillers in geopolymer mixtures without changing the composition of the main precursors and molar concentrations of activating solutions. Research has shown that it is not justified to replace reactive ingredients; it is more effective to replace a non-reactive filler, such as sand. The reactive silica contained in the diatomite has an additional positive effect on the formation of the geopolymer structure, as indicated by an increase in compressive strength of up to 24%.

## 2. Materials and Methods

### 2.1. Materials

Geopolymers were made based on two reactive material—fly ash and metakaolin. As a fine aggregate, quartz sand was used. Fly ash came from the heat and power plant in Skawina (Poland), and metakaolin came from the Czech Republic (Keramost, Kadaň, Czech Republic). Both materials were previously used for geopolymer manufacturing, because of their favorable chemical composition [37].

The diatomite came from the Jawornik Ruski deposit (Jawornik Ruski, Poland). This diatomite contains about 80–90% SiO_2_, so it should be useful as a sand substitute in building materials. The data about chemical composition came from previous research on the same material [8,9]. It was confirmed using EDX analysis. The obtained diatomite, as a by-product from the processing of diatomite, had a grain size of max. 0.063 mm, according to the supplier’s declaration. To confirm this declaration, suitable research was conducted and the findings are presented in the Results section.

**Figure 1:**
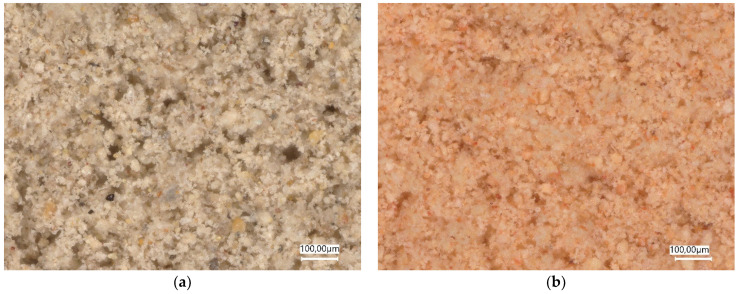
Diatomite (**a**) without any pre-treatment—“as delivered”, magnification 300× and (**b**) after calcination at 850 °C, magnification 300×.

For the planned composites, the diatomite was prepared in two forms: (a) without any pre-treatment—“as delivered”—DAD and (b) after calcination at 850 °C—DC (Figure 1). The calcination temperature was selected based on previous experiences. Calcination was achieved at 4 h.

### 2.2. Sample Preparation

**Figure 2:**
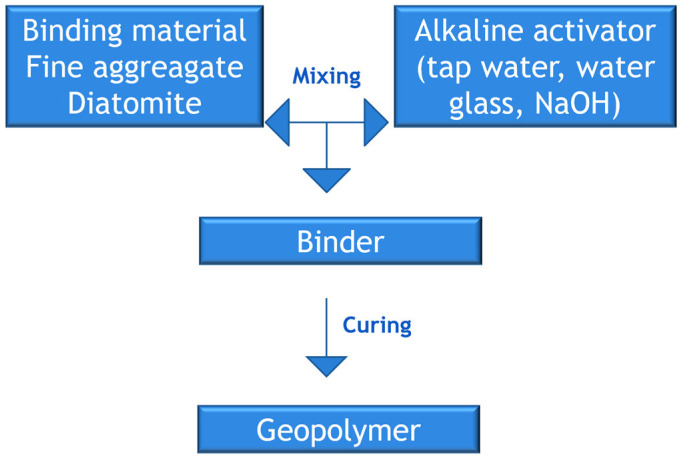
Diagram for sample preparation.

The samples were prepared according to the diagram presented in Figure 2. 

At the first stem, dry ingredients were mixed and an alkaline activator was prepared. The alkaline solution consisted of technical sodium hydroxide flakes with aqueous sodium silicate (a ratio of 1:2.5 was used) and tap water. As a result of mixing, a 10 M solution was obtained. Next, dry and liquid ingredients were mixed to obtain a homogenous paste—about 15 min. The obtained geopolymer mass was filled with a set of 50 mm × 50 mm × 50 mm cubic forms. To remove the air from the paste, the sets were placed on a vibrating table for about 5 min. Then, the sets were covered with a layer of polyethylene film (to prevent rapid moisture loss) and placed in a laboratory dryer for 24 h at a temperature of 75 °C. The temperature was selected based on previous experience. After this time, the samples were removed from the molds and cured in the laboratory. 

The fly ash and metakaolin in a weight ratio of 50/50% (reference samples) and with 1% and 3% additions of diatomite (as a replacement for the sand) were prepared. Diatomite was applied in two forms: “as delivered” and after calcination at 850 °C. The composition of prepared samples is presented in Table 1.

The compressive strength was investigated after curing at 14 and 28 days.

For clarity, to distinguish between the second batch of tests related to replacing part of the sand with diatomite, Table 2 was prepared, which gives the compositions of the prepared geopolymer mixtures.

### 2.3. Methods

Particle size analysis was carried out using a wet method on a Particle Size Analyzer (AntonPaar GmbH, Graz, Austria). The oxide composition was investigated using an EDX-7200 device.

Microscopy pictures were obtained using Keyence VHX-7000 (Osaka, Japan). The microstructure was investigated using scanning electron microscopy: JEOL JSM-IT200 with an energy dispersion X-ray spectroscopy system (Tokyo, Japan). The investigated material was covered with a gold layer using a DII-29030SCTR Smart Coater before observation to ensure good conductivity.

The bulk density in a loose and compacted state was measured for aggregates with a grain size of up to 63 mm, according to the standard PN-EN 1097-3:2000: tests for mechanical and physical properties of aggregates [38]. It was determined for calcinated and non-calcinated diatomite. The test was conducted on a standardized measuring cylinder made of stainless steel with a capacity corresponding to the size of max. aggregate grains: grain size up to 4 mm—the capacity of the container was 1 L. Three aggregate samples were prepared and dried at 110 °C to constant weight. The weight of the sample was 120–150% of the weight needed to fill the container.

Calorimetric measurements of diatomite were performed using thermogravimetry (TG) combined in one process with differential scanning calorimetry (DSC) and quadrupole mass spectrometry (QMS). The test was conducted on an STA 409CD NETZSCH in temperatures up to 1200 °C (Differential Thermal Analysis (DTA), coupled with Thermogravimetry (TG), was performed with NETZSCH STA 409 C/CD instrument (Netzsch GmBH, Selb, Germany)). An argon atmosphere was used, with a heating and cooling rate of 10 °C/min.

The compressive strength of the geopolymer composites was determined after 14 and 28 days. It was tested on 50 mm × 50 mm × 50 mm cubic samples using a MATEST 3000 kN test machine (Arcore, Italy) at a speed of 0.05 MPa/s according to EN 12390-3: testing of hardened concrete [39]. For each series, a minimum of 3 samples were used. The obtained average values were rounded to integers. Statistical analysis of the results allowed us to obtain calculations of the standard deviations, presented as error bars.

## 3. Results

### 3.1. Properties of Diatomite

To obtain accurate results, the measurements taken using a Particle Size Analyzer were collect for five samples (Table 3).

**Figure 3:**
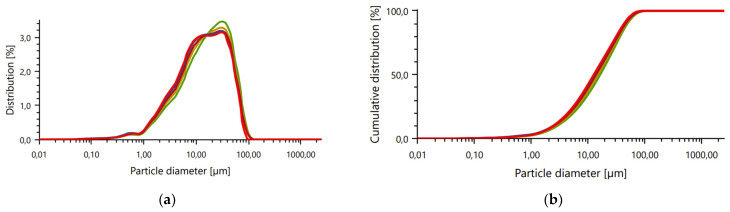
Particle size analysis of diatomite: (**a**) distribution (volume); (**b**) cumulative distribution (volume)—undersize.

The obtained values for the particle’s distribution are presented in Figure 3. 

The analysis confirms that the particle size for most particles (D_90_) was under 0.063 mm (mean value 45.688 µm). The mean value for the samples was 20.731 µm.

Additionally, calcinated and non-calcinated diatomite oxide compositions were provided. The results are presented in Table 4.

The provided EDX analysis confirms the declaration about diatomite composition from previous research [8,9]. The main element of the composition is SiO_2_. The second element is Al_2_O_3_. These two elements are favorable, taking into consideration application in the geopolymerization process [4]. The calcination process does not significantly influence oxide composition.

**Figure 4:**
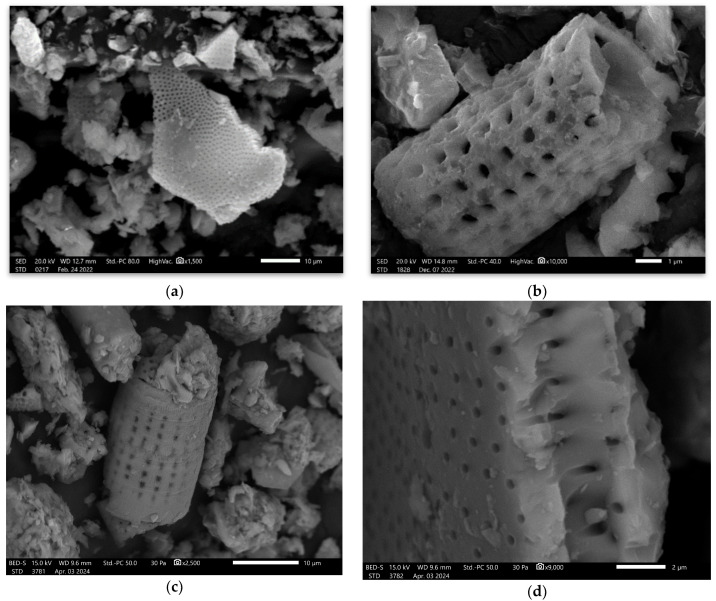
SEM images of diatomite: (**a**) magnification 1500×; (**b**) magnification 10,000×; (**c**) magnification 2500×; and (**d**) magnification 9000×.

In the next step, scanning electron microscopy (SEM) observations were made—Figure 4. During the SEM investigation, typical structures for diatomite were observed. These structures are connected with the material origin, which is fossil microorganisms [40]. The structures are mainly built from silica.

The results from material bulk density measurements are presented in Table 5.

A significant difference was observed in the reduction of bulk density in both loose and compacted states. Based on this result and previous research reported in the literature [23,26], it was supposed that there should be a huge difference between using diatomite as delivered and in its calcinated form. However, the compressive strength test does not confirm this expectation.

**Figure 5:**
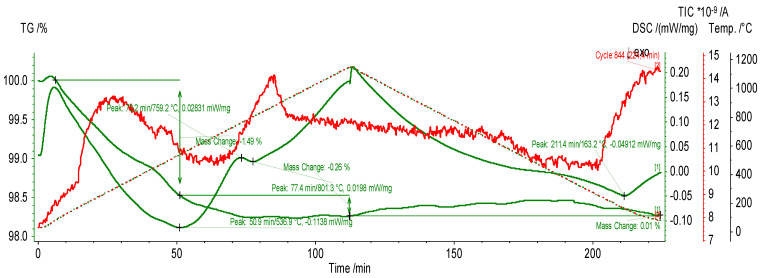
TGA/DTG and DSC analysis of diatomite.

TG/DTG analysis indicates thermal decomposition processes and gives information about the rate of mass change concerning temperature, which can include information about specific decomposition or phase transition processes occurring during sample heating. Additionally, DSC results provide information about energy values connected with thermal transformations (Figure 5).

The thermal analysis results show the main changes during heating at temperatures 536.9 °C, 759.2 °C, and 801.3 °C and also during cooling at temperature 163.2 °C. The temperature of 536.9 °C is probably connected with the mass loss process as a result of the degradation of organic components. From the point of view of chemical composition the content of silica should increase after this process as a result of removing hydroxyl groups and other organic substances [20]. There is an observed phase transition at higher temperatures, such as 759.2 °C and 801.3 °C. The mechanism of these changes is probably connected with the crystallization of the amorphous phase into quartz and cristobalite [20]. Up to this temperature, the weight loss slowly continued. This is probably connected with the removal of bound water as a result of the decomposition of the structure of diatomite [20]. The temperature of calcination was selected as 850 °C. This temperature is above the last phase transition and obtains a uniform material structure with a crystalline phase. This was possible because the aim of the addition of diatomite was not connected with obtaining the additional amount of silica in the alkali solution, for which an amorphous state is required [20,23,27].

### 3.2. Mechanical Properties of Composite

**Figure 6:**
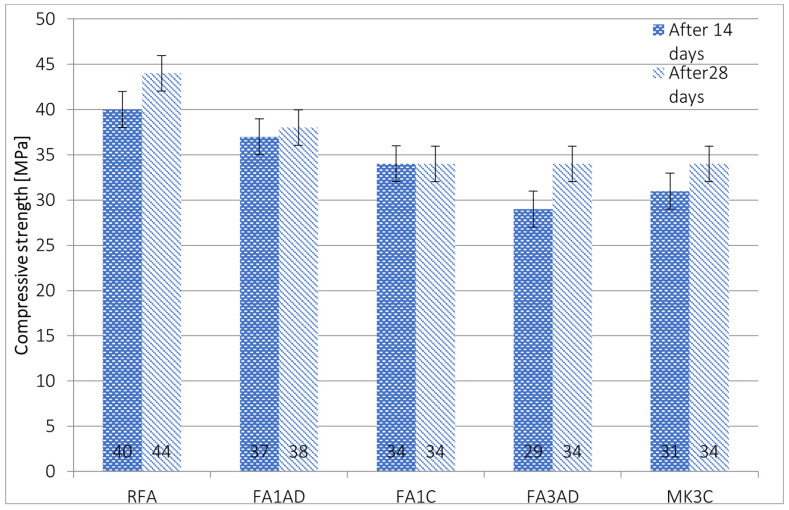
Results of the compressive strength of fly-ash-based geopolymers.

**Figure 7:**
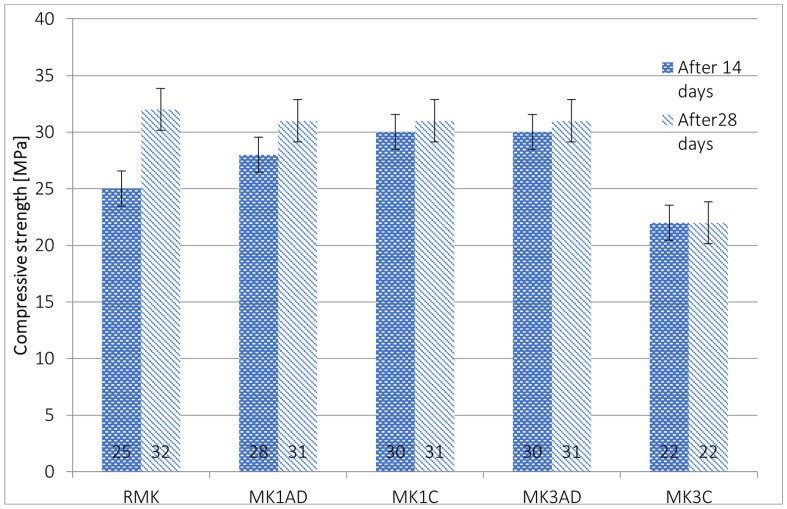
Results of the compressive strength of metakaolin-based geopolymers.

Mechanical strength was investigated on the samples cured for 14 and 28 days to observe the behavior of the samples over time. The expected mechanism was the reinforcement of geopolymer samples, which was reported in the literature previously [41]. Most of the samples behave according to this prediction (Figure 6 and Figure 7).

Higher compressive strength values were obtained for fly-ash-based geopolymers (Figure 6).

The addition of diatomite in each case caused a reduction in compressive strength in the case of fly-ash-based materials. The reduction was between 8 and 28% for samples investigated after 14 days and between 14 and 24% for the samples investigated after 28 days, respectively. The large amount of diatomite caused a higher reduction in compressive strength. A reduction in compressive strength as an effect of diatomite addition has been observed previously for geopolymers based on fly ash [12,16]. However, other investigation showed that diatomite addition increased mechanical properties [32]. 

The behavior against time was consistent with the prediction. The mechanical strength increased or stayed at the same level in all cases. The most visible rise in compressive strength occurred for the composite with 3% non-calcinated diatomite addition—approximately 15%. 

Better results were obtained for non-calcinated diatomite compared to calcinated. It is clearly visible for the 1% addition of diatomite. This kind of behavior is not coherent with results previously reported in the literature [23,26], but, taking into consideration the practical point of view, it results in higher profitability of the disposal process of industrial by-products (diatomite fume). 

The metakaolin-based samples show slightly different results for the compressive strength test—Figure 7.

In most cases, the addition of diatomite to the metakaolin matrix caused increasing compressive strength compared to the reference material in the short term—14 days—and decreased the compressive strength over a longer period—28 days. The values obtained for 1% diatomite were 12% higher for non-calcinated diatomite and 20% higher for calcinated diatomite, respectively. In the case of 3% diatomite addition, for the non-calcinated material, the compressive strength was noted to increase by about 20%, but for calcinated material, there was a drop of about 12%. For the 28-day period, diatomite addition caused a reduction in mechanical strength off between 3 and 31%. It is worth noting that previous research reported in the literature shows different behavior-increasing mechanical properties for metakaolin-based geopolymers [15,20,21].

The behavior over time was similar to fly-ash-based samples. Over time, the value of compressive strength increased. However, the increases were not as pronounced as for fly-ash-based materials.

For the 1% diatomite addition, the process of calcination did not influence the final value of compressive strength after 28 days. In the case of 3% diatomite addition, the calcination process caused deterioration of these properties. The behavior in the metakaolin matrix confirms a lack of justification for implementing the calcination process for the investigated materials.

**Figure 8:**
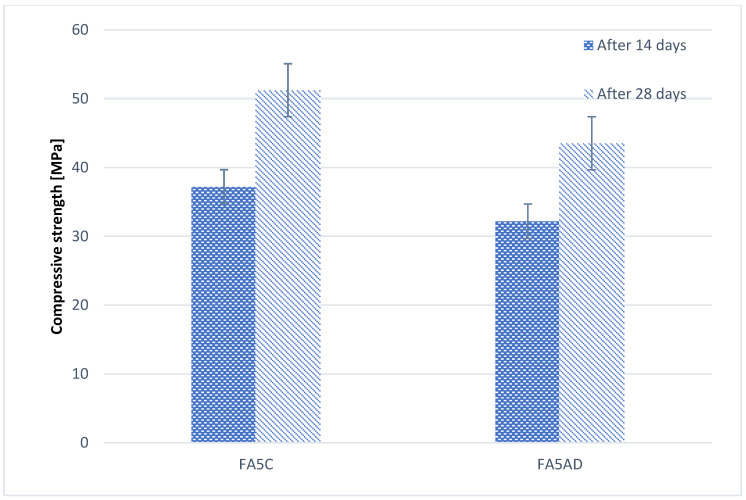
Results of the compressive strength of fly-ash-based geopolymer with a 5% addition of diatomite as a replace for sand.

Figure 8 shows the results of the compressive strength test for samples from the variant in which a 5% addition of diatomite was used to replace part of the sand. The chart shows the test results after 14 and 28 days of hardening and for two variants of the introduced diatomite: as delivered and calcined diatomite.

As shown in Figure 8, the addition of 5% diatomite during sand reduction increases the compressive strength compared to the reference sample (values shown in Figure 6). This is due to the fact that diatomite is not reactive and is not dissolved during the alkaline activation process, so it does not participate in the formation of the geopolymer structure but does serve as a filler. This is confirmed by the SEM images below in Section 3.3. The improvement in strength properties occurs here as a result of better adhesion between the geopolymer matrix and the diatomite particles than when using sand.

### 3.3. Microstructure

**Figure 9:**
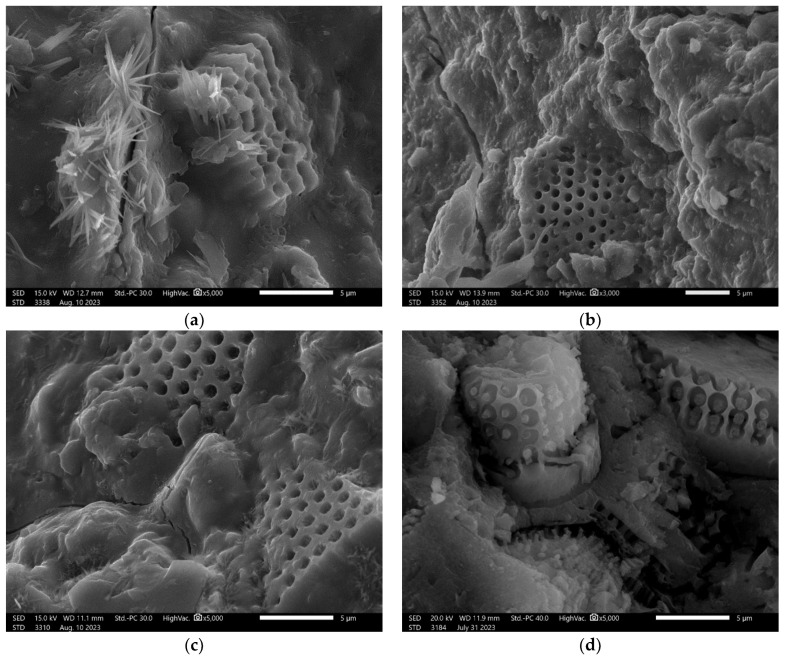
SEM images of geopolymer composites with diatomite: (**a**) magnification 5000×; (**b**) magnification 3000×; (**c**) magnification 5000×; and (**d**) magnification 5000×.

The provided SEM investigation for the composites allows us to confirm the good adhesion of the diatomite particles with the geopolymer matrix in both cases—fly-ash-based materials and metakaolin-based materials—Figure 9.

In Figure 9, good cohesion is visible between diatomite particles and the matrix. The characteristic structures of diatomite are built inside the matrix (Figure 9a–d). They were not dissolved. In the case of the cracking, they go through the matrix material and are not focused on the diatomite particles (Figure 9a–c). There is a lack of visible agglomeration of diatomite in the material. 

## 4. Discussion

The analysis of diatomite shows its suitability as an additive for geopolymer materials. The most interesting aspect of the undertaken works was thermal analysis. To compare the provided research with the literature, the most important aspects of the thermal properties are summarized in Table 6. It is worth noticing that, in previous publications, only a small number of investigations of this type have been conducted for diatomite. Today, research is connected mainly to TGA/DTG research of prepared compositions [20,23,27].

Previous research shows that the addition of diatomite improves the thermal properties of the composite while reducing its density and mechanical properties. The addition of calcinated diatomite has a stronger influence than non-calcinated. This observation is in line with some previous research [21]. Also, the temperature of calcination could influence the obtained values. Previously, this research was conducted experimentally by Thammarong et al. [26]. In that case, the maximum density and compressive strength were observed for geopolymers containing diatomite calcinated at 700 °C [26]. The thermal properties were not analyzed in that work [26]. The provided results pointed out that TGA/DTG analysis can support the process of optimization of the calcination temperature depending on the origin of the diatomite used. 

To compare the obtained results for geopolymer composites with the addition of diatomite, the publication that included similar research was selected and is listed in Table 7. The research that was taken into consideration is connected with solid geopolymers containing diatomite, with a focus on mechanical properties.

The above research shows the different behaviors of geopolymers with diatomite addition. This addition does not always increase the mechanical properties. In the presented research, the three most important factors were analyzed—the material of the matrix, pre-treatment of the diatomite, and their amount. The obtained results are not always in line with previous research; but, it must be noted that the previous research is also not always coherent between studies. The tested amount of diatomite in the literature is very different, with values ranging from below 1% [15] to 80% [14]. Nevertheless, a small amount of this additive seems to be more favorable than a very large amount. 

There could be several reasons for the decrease in geopolymer mechanical properties after the introduction of diatomite. Possible reasons could be connected with changes in the ratio between Si and Al; in particular, the higher content of Si can cause an imbalance between elements. As an effect of a lack of balance, improper bonding in the material can occur, which causes a reduction in mechanical properties [41,42]. An additional reason could be the appearance of silicate during the curing process, which is also caused by the high amount of Si. In this case, a proper geopolymer 3-dimensional structure with a theatrical network is not created and the chain in the material is relatively short. This also impacts the materials mechanical properties [43,44]. Other possible explanations such as aggregate agglomeration or a lack of coherence with the matrix were rejected based on SEM images.

The obtained results show a decrease in compressive strength, but the obtained values are still reasonable, thereby confirming the suitability of its application in the construction industry, as well as in other areas [3,45]. In this industry, applications close to the place of diatomite fume production confer the greatest advantages, because of the reduced cost of transportation. This kind of application can be connected with infrastructure dedicated to transportation in mining, such as pavements. Despite the decrease in mechanical properties, the material can be used for some construction applications, such as pavements. The obtained materials have reasonable compressive strength and can be utilized “on-site” near the source of waste production. From an economic point of view, especially important is the minimal influence of the calcination process for such additions; this lowers the cost of final materials and their environmental influence through a reduction in the energy necessary for processing.

## 5. Conclusions

The conducted research indicates the possibility of using diatomite waste in geopolymer materials. In the first stage, the synthesis of geopolymers was carried out using diatomite with a grain size of 0–0.063 mm in amounts of 1% and 3%, using two types of matrices based on fly ash and sand or metakaolin and sand in a mass ratio of 50/50%. The addition of diatomite was tested under delivery conditions and after calcination at a temperature of 850 °C. Based on the results obtained, the following conclusion can be formulated:

- Studies of the microstructure of non-calcined diatomite confirmed its typical structure and identified elements of fossil diatoms. Particle size analyses and SEM observations confirmed the fine particle size of the diatomaceous earth used.
- The bulk density of the diatomite after calcination decreases significantly.
- The selected calcination temperature of 850 °C is correct, as confirmed using thermal analysis.
- The addition of 1 and 3% diatomite to geopolymers based on fly ash resulted in a decrease in their mechanical properties.
- The addition of 1 and 3% diatomite to metakaolin-based geopolymers increased their mechanical properties in the early stage (after 14 days), but, in the long term, it worsened the final properties.

The next stage of the research consisting of the introduction of 5% of diatomite, partially replacing the amount of sand (but without changing the amount of binder), showed that this variant of introducing diatomite is more beneficial and an improvement in the parameters of geopolymers was observed. It was observed that a greater improvement in properties was achieved in the case of the addition of calcined diatomite, so such treatment is justified.

Diatomites, despite their high silica content, are not a material that can be used as a precursor of geopolymerization but should be treated as a replacement for popular fillers such as sand. Using them in this way improves the strength of geopolymers compared to compositions without the addition of diatomite.

## Figures and Tables

**Table 1:** Determination of samples with the addition of 1 and 3% of diatomite as a substitute for fly ash and metakaolin.

No	Metakaolin [g]	Fly Ash [g]	Sand [g]	Diatomite [g]	Solution	Comment
RMK	600	0	600	0	300 mL sodium hydroxide and sodium silicate; 10 M	Reference sample
MK1AD	594	0	600	6		Diatomite “as delivered”
MK1C	594	0	600	6		Diatomite after calcination
MK3AD	582	0	600	18		Diatomite “as delivered”
MK3C	582	0	600	18		Diatomite after calcination
RFA	0	600	600	0		Reference sample
FA1AD	0	594	600	6		Diatomite “as delivered”
FA1C	0	594	600	6		Diatomite after calcination
FA3AD	0	582	600	18		Diatomite “as delivered”
FA3C	0	582	600	18		Diatomite after calcination

**Table 2:** Determination of samples with the addition of 5% diatomite as a substitute for the filler-sand.

No	Metakaolin [g]	Fly Ash [g]	Sand [g]	Diatomite [g]	Solution	Comment
FA5AD	0	600	570	30	300 mL sodium hydroxide and sodium silicate; 10 M	Diatomite “as delivered”
FA5C	0	600	570	30		Diatomite after calcination

**Table 3:** Particle size distribution of diatomite.

Serie	D₁₀ [µm]	D_50_ [µm]	D_90_ [µm]	Mean Size [µm]	Span
Sample 1	2.895	16.298	49.327	22.960	2.849
Sample 2	2.678	14.733	47.194	21.549	3.022
Sample 3	2.540	13.774	45.627	20.626	3.128
Sample 4	2.416	12.896	43.384	19.392	3.177
Sample 5	2.448	12.580	42.909	19.126	3.216
Mean value	2.595	14.056	45.688	20.731	3.078

**Table 4:** Oxide composition of diatomite.

Name of the Sample	SiO_2_	Al_2_O_3_	Fe_2_O_3_	K_2_O	TiO_2_	SO_3_	CaO	MnO	V_2_O_5_	ZrO_2_
DAD	80.620	13.248	3.200	1.693	0.419	0.412	0.310	0.026	0.025	0.010
DC	80.766	13.661	3.019	1.604	0.389	0.177	0.296	0.024	0.027	0.009
	**Cr_2_O_3_**	**ZnO**	**SrO**	**CuO**	**Ir_2_O_3_**	**Y_2_O_3_**	**NiO**	**PbO**	**NbO**	
DAD	0.008	0.006	0.005	0.005	0.004	0.003	0.002	0.002	0.001	
DC	0.008	0.006	0.005	-	0.004	0.003	0.002	-	0.002	

**Table 5:** Bulk density of diatomite.

	Diatomite as Deliver	Diatomite after Calcination
Bulk density—loose condition	0.72	0.35
Bulk density—compacted state	0.80	0.39

**Table 6:** Comparison of obtained results for TGA/DTG with the literature.

No.	Material Composition	Main Findings	Reference
1	Diatomite, Jawornik Ruski, Poland	The temperature of calcination was selected as 850 °C. This temperature is above the last phase transition and obtains a uniform material structure with a crystalline phase.	Current research
2	Metakaolin (MK) from Metacaulim do Brasil Ind. Com. and diatomite (D); activated using potassium hydroxide and potassium silicate	TGA can be applied to determine the exact optimum calcination temperature. The calcination temperature was optimized at 400 °C. Diatomite addition improves the mechanical properties and microstructure changes. Diatomite addition decreases the filtrate volume.	[20]
3	A total of 70% of sodium silicate, 8.65% of catalyst Na_2_SiF_6_, 21.3% of wt. silico-aluminate source (replaced by D), and 0.05% of Si powder; sodium silicate; foaming agent based on vegetable protein	DTG allows us to explain the behavior of different material compositions against the curing time. Increasing the amount of diatomite from 5% to 50% improves the mechanical properties and decreases the density of geopolymers. Above 50% of diatomite, the mechanical properties were deteriorated.	[23]
4	Diatomite with the addition of different % of wood biomass ash; dry activator (NaOH mixed with wood biomass)—one part geopolymer	The TG/DTG thermograms show the dilution of reactive aluminosilicate caused by the addition of high diatomite content. The performance of the prepared cement depends on the content of the dry activator and diatomite.	[27]

**Table 7:** Comparison of obtained results with the literature.

No.	Based Material	Diatomite	Activator	Diatomite Influence	Source
1	FA and MK	Amount (A): 1%, 3%; Origin (O): Poland (Jawornik Ruski); Treatment (T): calcinated and non-calcinated; Size (S): max. 0.063 mm	sodium hydroxide (SH), and sodium silicate (SS)	Possibility to use geopolymerization process as a method of processing diatomite fume (industrial by-product), despite the decreasing compressive strength (CS).	Current research
2	Lignite FA from Mae Moh power station, Thailand	A: 0 to 100%; O: Thailand (Lumpang); T: calcined at 800 °C for 6 h; S: Median particle size, d50 18.3 µm	SH and SS	Despite the improvement in workability, the CS and modulus of elasticity decreased. A weight reduction was observed.	[12,13]
3	Calcium aluminate cement (CAC)	A: 80%; O: South America	SH and a low-alkalinity solid activator (Na_2_SiO_4_)	Mixtures with diatomite do not create proper bonding in geopolimerization process and the material has very low mechanical properties.	[14]
4	MK	A: 0–1.5% S: 3–29 µm, average 14 µm	potassium hydroxide and potassium silicate	An improvement in the mechanical properties was noticed. The microstructure changed and the filtrate volume decreased.	[15]
5	Ultra-fine, amorphous MK	O: Turkey (Kizilcahamam); T: calcined at 400 °C temperature	potassium silicate solution was prepared using diatomite or silica fume (SF)	The four-point flexure strength of diatomite was lower than that of SF but the CS for diatomite was higher.	[20]
6	MK from Ranong province, Thailand	A: 5, 10, 15, 20, and 25%; O: Thailand (Lumpang)	SH and SS	The CS of the samples increased with increased amounts of diatomite up to 20% and then decreased.	[21]
7	MK from Ranong province, Thailand	O: Thailand (Lumpang); T: Calcination in temperatures 500–1200 °C for 2 h	SH and SS	Maximum density and CS were observed for geopolymers containing diatomite calcinated at 700 °C.	[26]
8	FA from Turkey, Class C	O: Turkey; S: average particle size of 1–5 µm with some 10 µm	SH	Diatomite allows a decrease in the amount of activator used and, at the same time, decreases the CS.	[16]
9	Red mud, blast furnace slag, and aggregate	Not specified. As an alternative to diatomite, SF was tested as a silica source.	SH and SS	The best results of CS were achieved for 10% of SF and 5% of diatomite.	[19]
10	FA	A: 1%, 2%, 3%, 4%, and 5%; O: Turkey: (Hırka district of Kayseri)	SH	A total of 1% and 2% diatomite substitution increased the flexural strength and CS compared to the reference sample.	[32]

## Data Availability

Data are contained within the article.
